# Successful rescue therapy with tenofovir in a patient with hepatic decompensation and adefovir resistant HBV mutant

**DOI:** 10.1186/1476-5926-5-1

**Published:** 2006-01-11

**Authors:** Vlad Ratziu, Vincent Thibault, Yves Benhamou, Thierry Poynard

**Affiliations:** 1 Service d'Hépatogastroenterologie, Hôpital Pitié Salpêtrière and Université Pierre et Marie Curie, Paris, France; 2 Laboratoire de Virologie, Hôpital Pitié Salpêtrière and Université Pierre et Marie Curie, Paris, France

## Abstract

**Background:**

Prolonged adefovir therapy exposes to the emergence of adefovir resistant hepatitis B virus mutants. Initial reports of the rtN236T mutation showed preserved sensitivity to lamivudine; however, complex mutations are emerging with reduced susceptibility to lamivudine.

**Case presentation:**

After 2 years of therapy, a cirrhotic patient developed the rtN236T and rtA181T adefovir resistant mutations. He had been previously treated with lamivudine, developed lamivudine resistance and, despite good compliance, had an incomplete response to adefovir. Adefovir resistance resulted in viral breakthrough with hepatitis flare-up and liver decompensation. Tenofovir had an excellent antiviral effect allowing sustained control of viral replication and reversal of hepatic failure.

**Conclusion:**

In patients with cirrhosis, adefovir resistance can lead to severe hepatitis. Tenofovir appears to be an effective treatment of adefovir resistant mutants. Incomplete control of viral replication with adefovir requires monitoring for viral resistance and should prompt a change in antiviral treatment.

## Background

In chronic hepatitis B, prolonged antiviral therapy with nucleoside analogues, such as lamivudine, is often necessary but may result in the emergence of escape mutants which can be detected in as many as 70% of patients, after 4 years of therapy [1]. Adefovir dipivoxil has potent antiviral activity on lamivudine-resistant hepatitis B virus (HBV) strains [2,3]; nonetheless, long term use of this nucleotide analogue is also associated with resistance in up to 18% of patients, after 4 years of therapy [4]. In the few cases published thus far, adefovir-resistant HBV displayed the rtN236T mutation and maintained susceptibility to lamivudine *in vivo *[5,6]. However, new adefovir-resistant mutations have been reported, such as the rtA181V mutation which is closely located to the rtL180M mutation conferring resistance to lamivudine [4]. It is uncertain whether those new adefovir escape mutants are susceptible to lamivudine [7] and thus drugs active on both adefovir and lamivudine-resistant HBV strains are needed.

We report on the case of a patient with HBV-related cirrhosis and lamivudine-resistant HBV who developed adefovir resistance with viral breakthrough and liver failure. The patient had both an rtN236T and a rare rtA181T mutation. Tenofovir fumarate was highly effective in controlling viral replication and reversing clinical symptoms.

## Case presentation

In 1997, a 55 year-old Vietnamese man was diagnosed with HBV cirrhosis without hepatitis delta virus (HDV), hepatitis C virus (HCV) or human immunodeficiency virus (HIV) coinfections. Probably, the route of infection was vertical transmission. Cirrhosis was confirmed by liver biopsy and was complicated only by grade 1 oesophageal varices (Child Pugh score A5). HBe antigen was negative, HBe antibody positive, HBV viral load (HBV-DNA) was 7.15 log_10 _copies/ml (Digene hybrid capture assay) and the HBV genotype was B. Alanine aminotransferase (ALT) values were twice the upper limit of normal (ULN).

In August 1998, lamivudine, 100 mg/day, was started. Three months later, HBV-DNA was undetectable by Digene assay and, in February 1999, by qualitative PCR (sensitivity limit at 4 log_10_). Lamivudine was inadvertently stopped after only 10 months of treatment, and then started again in January 2000 when a relapse occurred with a rising HBV-DNA to 5.75 log_10 _and ALT values at 3.1 ULN. The next 2 years of treatment were uneventful with undetectable HBV-DNA by quantitative PCR (cut-off at 2.3 log_10_, MONITOR COBAS, Roche) and normal ALT values.

In January 2002, after 24 months of uninterrupted lamivudine treatment, HBV-DNA became detectable by PCR (2.6 log_10_), and, in June 2002, a viral breakthrough was documented when HBV-DNA rose to 9.2 log_10 _with ALT at 2.7 ULN. The patient was asymptomatic. Sequencing of the HBV polymerase revealed two common lamivudine resistant mutations – rtL180M and rtM204V. Adefovir dipivoxil, 10 mg/day, was added to the ongoing lamivudine treatment. The evolution of HBV viral load is shown in the Figure. Over the next 10 months, HBV-DNA never fell below 5 log_10_. In June 2003, the HBV-DNA titer was 5.28 log_10_. Lamivudine was then stopped, but adefovir was maintained; in addition, pegylated interferon α-2a was introduced (180 μg/week). In August 2003, and after five injections, the patient decided to stop pegylated interferon administration due to poor drug tolerance: anorexia, dry mouth, diarrhoea, weight loss and back pain. Thrombopenia was also detected.

After 4 months of uninterrupted adefovir monotherapy, HBV-DNA was still at 4.9 log_10 _and the same lamivudine resistant mutations were detected without adefovir resistant mutations. In September 2004, while on adefovir monotherapy, and 15 months after having stopped lamivudine and 13 months after having stopped pegylated interferon, HBV-DNA rose to 8.3 log_10_, ALT to 10 ULN and aspartate aminotransferase to 7 ULN. Prothrombin time was 78%, and total bilirubin and serum albumin were normal. Two adefovir resistant mutations, rtA181T and rtN236T, were detected, whereas previous lamivudine resistant mutations, rtL180M and rtM204V, were absent. Anti-HDV and anti-HCV antibodies detection remained negative.

A test trial of a higher dose of adefovir (20 mg/day) was started. One month later, HBV-DNA was at 7 log_10 _and ALT levels remained unchanged. The patient's condition deteriorated with the occurrence of oedema, ascites, jaundice, mild renal insufficiency (serum creatinine 110 μmol/l) and decreased prothrombin time (47% of normal). Tenofovir fumarate was then started at a dose of 300 mg/day, while adefovir was continued for one month at 10 mg/day and then stopped. Two months later, HBV-DNA became undetectable by PCR, prothrombin time rose to 90% while jaundice and ascites resolved. HBV-DNA was still undetectable by PCR after 8 months of tenofovir treatment, on three separate occasions. The patient is currently listed for liver transplantation.

**Figure 1:**
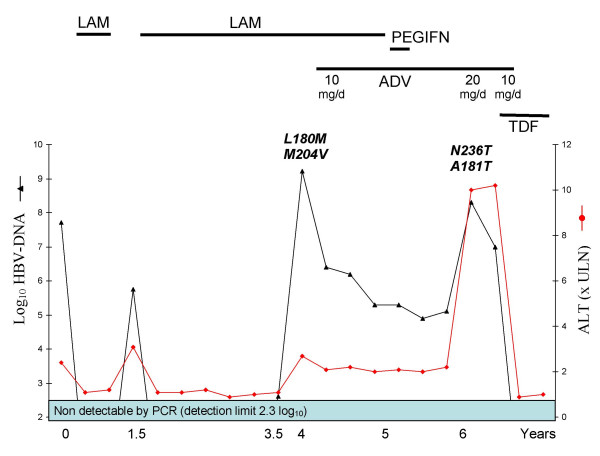
Virological and biochemical course before and after the emergence of adefovir resistant mutations. LAM: lamivudine; ADV: adefovir dipivoxil; TDF: tenofovir disoproxil fumarate.

## Discussion

Despite good compliance, after developing lamivudine resistance the patient did not respond to adefovir dipivoxil treatment. Although the reasons are unclear, primary non-response to adefovir has been described in 8% to 15% of HIV-negative [2,3] and HIV-positive [8] patients infected with lamivudine-resistant HBV. Incomplete viral suppression could have contributed to the emergence of adefovir resistant mutations. Indeed, it has already been shown that a high viral load (>3 log_10_) after 6 months of lamivudine therapy predicted the emergence of resistance [9,10]. The patient developed both rtN236T and rtA181T mutations while under adefovir treatment for 2 years. The rtN236T and rtA181V are well-described adefovir resistant mutations [5,6,11], whereas the rtA181T is very rare and has only been described in 1 out of 22 patients with adefovir resistance, after 4 years of treatment [4]. Viral breakthrough was associated with severe hepatic decompensation, probably favoured by longstanding underlying cirrhosis. Similar to lamivudine resistance, clinicians should be aware of the possibility of severe hepatic decompensation in cirrhotic patients developing adefovir resistance; therefore, clinicians should implement viral resistance monitoring strategies.

Non-randomized studies suggested that tenofovir has a more potent antiviral activity than adefovir on lamivudine-resistant HBV [12], whereas in HIV-HBV patients with primary non-response to adefovir, tenofovir is highly effective [8]. Because of the emergence of the rtA181T mutation (which is closely located to codon 180), conferring resistance to lamivudine and closely related to rtA181V, we chose to treat this patient with tenofovir rather than with lamivudine. In fact, the rtA181V mutation reduces the susceptibility to lamivudine 14 fold *in vitro*, while *in vivo *lamivudine has only a limited antiviral effect on that mutant strain [13]. Brunelle *et al. *[14] reported recently decreased *in vitro *susceptibility to both tenofovir and adefovir of two HBV resistant mutants (N236T adefovir and L180M + M204V + N236T lamivudine/adefovir resistant strains). A similar fold resistance over wild-type HBV was reported for tenofovir and adefovir (4.5, and 3.2 to 6.4 fold, respectively), suggesting a marginal benefit of tenofovir over adefovir. However, one might question the *in vivo *relevance of those *in vitro *experiments. In this patient with the double rtN236T and rtA181T mutations, the antiviral response to tenofovir was excellent. A rapid and sustained inhibition of viral replication, by sensitive PCR, was detected after only 2 months of treatment and resulted in an improvement in liver function to a degree that could not be anticipated from *in vitro *resistance studies. Discrepancies between *in vitro *drug susceptibility and *in vivo *findings could be related to differences in pharmacodynamics between the two drugs.

## Conclusion

Adefovir resistant mutations can induce severe hepatic decompensation in cirrhotic patients and can be preceded by incomplete viral suppression. In these patients close viral monitoring is mandatory. For avoiding the emergence of resistant mutants, incomplete viral response to adefovir should prompt a change in antiviral treatment. Tenofovir appears to be an effective treatment of adefovir resistant mutants.

## Competing interests

The author(s) declare that they have no competing interests.

## Authors' contributions

Vlad Ratziu provided clinical care for the patient, and wrote the manuscript. Vincent Thibault carried out the virological analyses, helped interpret the data and participated in the writing of the manuscript. Yves Benhamou and Thierry Poynard helped interpret the data and critically revised the manuscript. All authors read and approved the final manuscript.
